# Dynamic changes of blood glucose level during capivasertib treatment in metastatic breast cancer: a case report

**DOI:** 10.1007/s13340-025-00841-x

**Published:** 2025-08-06

**Authors:** Shinsuke Sasada, Haruya Ohno, Hisae Taonouchi, Marino Teshima, Momoko Takaya, Mutsumi Fujimoto, Haruka Ikejiri, Ai Amioka, Hideo Shigematsu, Morihito Okada

**Affiliations:** 1 Department of Surgical Oncology, Research Institute for Radiation Biology and Medicine, Hiroshima University, 1-2-3 Kasumi, Minami-Ku, Hiroshima City, Hiroshima 734-8551 Japan; 2 Department of Breast Surgery, Hiroshima University Hospital, Hiroshima, 734-8551 Japan; 3 Endocrinology and Diabetic Medicine, Hiroshima University Hospital, Hiroshima, 734-8551 Japan

**Keywords:** Breast cancer, AKT inhibitor, Capivasertib, Continuous glucose monitoring, Hyperglycemia

## Abstract

Hyperglycemia is a well-recognized adverse event associated with capivasertib, an oral AKT inhibitor, and may occasionally progress to serious conditions such as diabetic ketoacidosis. We report the case of a 73-year-old woman with hormone receptor-positive/human epidermal growth factor receptor 2-negative metastatic breast cancer who developed marked hyperglycemia shortly after initiating capivasertib therapy. Marked hyperglycemia was observed on day 11 of treatment, despite normal baseline levels. Capivasertib was temporarily discontinued, and insulin therapy was initiated. Continuous glucose monitoring revealed transient hyperglycemic episodes occurring exclusively on dosing days, which resolved within approximately 12 h. This pattern suggests a pharmacodynamic correlation and reversible effect of capivasertib on glucose metabolism. Rapid-acting insulin was required only on days when capivasertib was administered. Notably, standard hemoglobin A1c monitoring failed to capture these fluctuations. This case underscores the importance of real-time glucose monitoring and individualized glycemic management during capivasertib therapy, particularly in patients with metabolic risk factors. Early detection and tailored intervention may help prevent severe hyperglycemic complications and facilitate the safe continuation of treatment.

## Introduction

Capivasertib, an oral pan-AKT inhibitor, when combined with fulvestrant, extends progression-free survival in hormone receptor (HR)-positive/human epidermal growth factor receptor 2 (HER2)-negative advanced breast cancer with disease progression on or after aromatase inhibitor-based therapy, especially in patients with AKT pathway alterations [1]. Hyperglycemia is a major adverse event associated with capivasertib. In the CAPItello-291 trial, patients with diabetes undergoing insulin treatment or with a hemoglobin A1c (HbA1c) level of 8.0% or higher were excluded, and the incidence of grade 3/4 hyperglycemic events was 2.3%. Although serious hyperglycemia was rare (0.8%), the median time to onset of hyperglycemia with capivasertib was 15 days; moreover, instances of diabetic ketoacidosis (DKA) have been reported [2–4]. A fatal case of DKA occurring in the early phase of capivasertib treatment has been reported in Japan. Consequently, the Japan Diabetes Society and the Japan Breast Cancer Society issued a warning on April 15, 2025, regarding the occurrence of hyperglycemia and DKA associated with capivasertib. Although obesity, a history of diabetes, and baseline blood glucose levels in the diabetic or pre-diabetic range have been identified as risk factors for severe hyperglycemia, the optimal strategy for glycemic control remains unclear [2]. We present a case characterized by distinctive glycemic fluctuations during capivasertib treatment, providing important insights into glucose monitoring and management in this setting.

## Case report

A 73-year-old woman with HR-positive/HER2-negative metastatic breast cancer was treated with fulvestrant and capivasertib. She had undergone breast-conserving surgery and axillary dissection for T2N1M0 left breast cancer 20 years previously, followed by adjuvant anthracycline- and taxane-based chemotherapy, whole breast irradiation, and endocrine therapy. The disease recurred with bone metastasis 6 years after surgery, and she was subsequently treated with seven endocrine agents, two CDK4/6 inhibitors, a mammalian target of rapamycin (mTOR) inhibitor, two chemotherapy regimens, and radiation therapy as appropriate. The breast cancer had spread to bone, liver, and spleen, and Guardant360 CDx assay (Guardant Health, Palo Alto, CA) revealed *PIK3CA* H1047R. She had a history of hyperlipidemia, her body mass index was 18.8 kg/m^2^, and her baseline fasting blood glucose level was normal (109 mg/dL) before capivasertib administration. She had no personal or family history of diabetes, and HbA1c was not measured before initiating capivasertib.

**Fig. 1:**
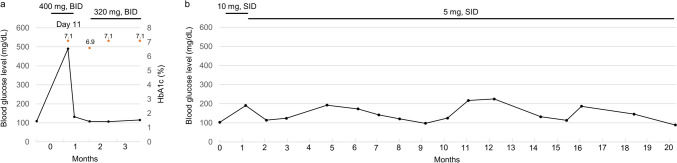
Blood glucose levels at the time of visits during capivasertib (**a**) and everolimus (**b**) treatment. Orange circles denote HbA1c levels. *BID* bis in die, *SID* semel in die

**Fig. 2:**
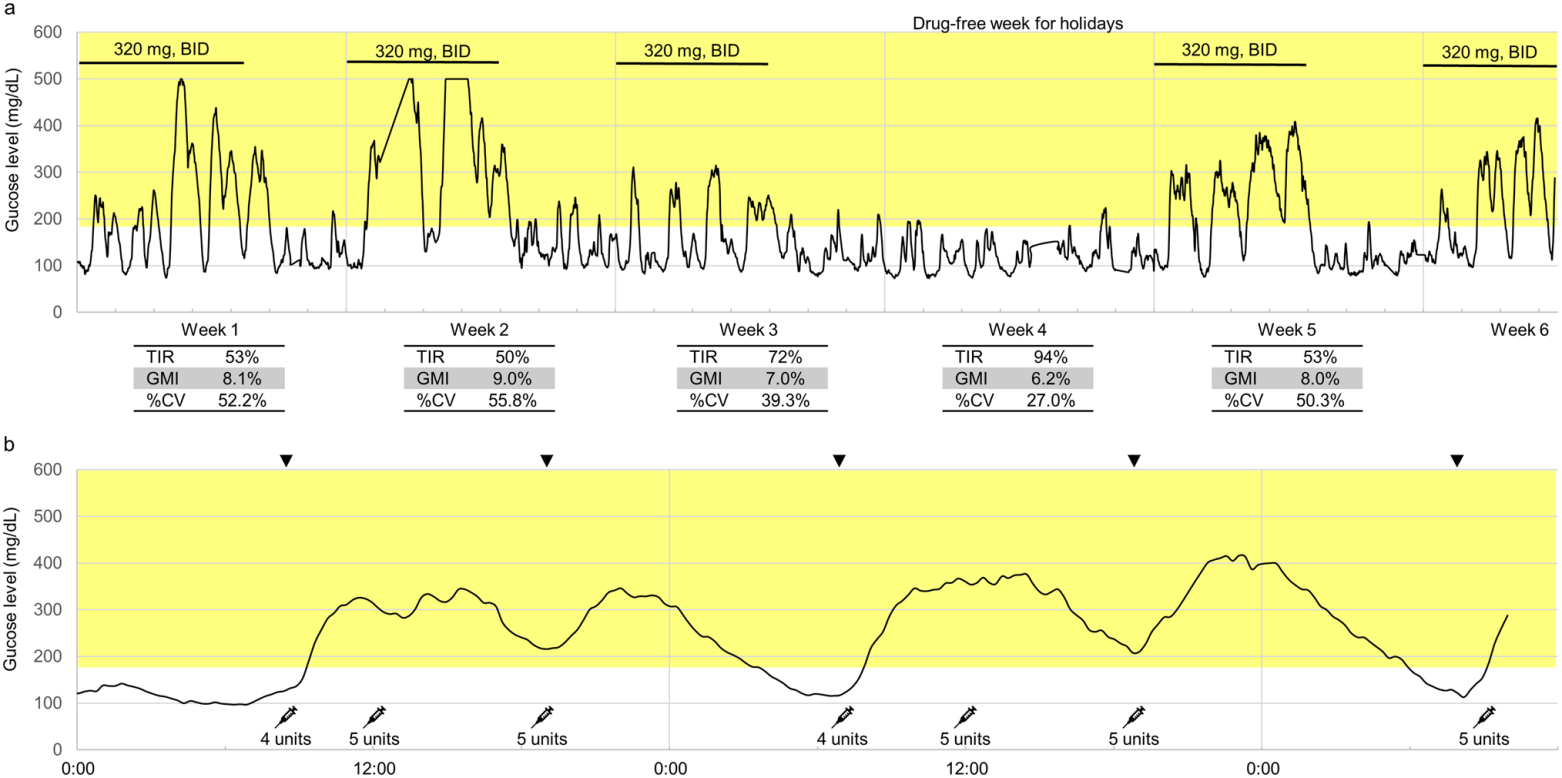
**a** Intermittently scanned continuous glucose monitoring for 6 weeks during capivasertib treatment, including weekly metrics: TIR, GMI, and %CV. **b** Intermittently scanned continuous glucose monitoring for two consecutive days [week 6 in **a**] during capivasertib treatment. Arrowheads denote the oral administration of capivasertib 320 mg. Syringe icons denote the rapid-acting insulin injection. The yellow-shaded area indicates glucose levels ≥ 180 mg/dL. *BID* bis in die, *%CV* coefficient of variation, *GMI* glucose management indicator, *TIR* time in range

On day 11 following initiation of capivasertib (400 mg twice daily for 4 days, followed by 3 days off), she was diagnosed with marked hyperglycemia (> 500 mg/dL) at her home clinic, despite being asymptomatic. She promptly sought medical attention, at which time her blood glucose level was 490 mg/dL, HbA1c was 7.1%, and urinary ketones were negative (Fig. 1a). She was referred to a diabetologist, capivasertib was temporarily withheld, and insulin therapy was initiated. Her blood glucose levels improved rapidly, and capivasertib was resumed at a reduced dose (320 mg twice daily, 4 days on/3 days off), accompanied by the initiation of FreeStyle Libre Pro^®^, an intermittently scanned continuous glucose monitoring (isCGM) system (Abbott Diabetes Care, Chicago, IL). isCGM revealed that hyperglycemia occurred exclusively on days when capivasertib was administered (Fig. 2a). Rapid-acting insulin was administered only on dosing days, with a baseline dose of 11 units per day divided into three administrations and supplemental doses adjusted according to a sliding scale. A representative 2-day is CGM trace demonstrated a characteristic rise in glucose levels 1–2 h after capivasertib intake, with levels returning to baseline approximately 12 h later (Fig. 2b). Thereafter, HbA1c remained stable, and blood glucose levels were within the normal range on day 1 of each treatment cycle (Fig. 1a). Mild glycemic fluctuations had been observed during prior everolimus treatment (Fig. 1b).

## Discussion

This report documents marked fluctuations in glucose levels during treatment with capivasertib and fulvestrant in a patient with metastatic breast cancer and hyperlipidemia.

Hyperglycemia is a common adverse event associated with capivasertib, following diarrhea, skin rash, nausea, fatigue, vomiting, headache, and decreased appetite in frequency. It typically occurs early in the course of treatment and can, in some cases, progress to a serious condition known as DKA. AKT signaling induces glucose transporter 4-mediated glucose transport into skeletal muscle and adipose tissue [5, 6]. In addition, AKT phosphorylates glycogen synthase kinase 3 (GSK3), and the resulting inactivation of GSK3 relieves its inhibition of glycogen synthase, thereby enhancing glycogen synthesis [7]. Therefore, inhibition of the phosphoinositide 3-kinase (PI3K)/AKT/mTOR pathway can lead to acute hyperglycemia. The role of AKT1/2 signaling in insulin secretion by pancreatic *β* cells remains controversial. AKT phosphorylation enhances calcium release from the endoplasmic reticulum and upregulates SNARE protein expression, both of which facilitate insulin secretion. Conversely, the activation of the insulin receptor/insulin receptor substrate/PI3K signaling cascade induces hyperpolarization of membrane K_ATP_ channels, thereby suppressing calcium-dependent insulin release [8]. Interestingly, short-term inhibition of the PI3K/AKT pathway has been reported to promote the exocytosis of newcomer granules in pancreatic *β* cells [9]. Regardless of the pathway involved, peripheral insulin resistance leads to compensatory hyperinsulinemia, which contributes to the gradual deterioration of *β*-cell function [10].

Alpelisib, an oral PI3Kα inhibitor, caused grade 3/4 hyperglycemia in 36.6% of patients in the SOLAR-1 trial [11]. Risk factors for hyperglycemia included diabetic or prediabetic glycemic status at baseline, body mass index ≥ 30 kg/m^2^, and age ≥ 75 years [12]. Cases of DKA associated with alpelisib have also been reported, and the reporting odds ratio for DKA events with alpelisib, based on an analysis of the U.S. Food and Drug Administration Adverse Event Reporting System pharmacovigilance database, was 9.84 [13–15]. In response, an algorithm for the monitoring and management of alpelisib-induced hyperglycemia has been proposed [5]. After initiating alpelisib, blood glucose should be monitored at least once every 2 weeks, then monthly, with HbA1c assessed every 3 months. Closer monitoring is recommended for patients at high risk of hyperglycemia. The recommended initial therapy is metformin, followed by pioglitazone, SGLT2 inhibitors, and insulin as needed.

Everolimus, an oral mTOR inhibitor, is associated with hyperglycemia. In the BOLERO-2 trial, grade 3/4 hyperglycemia occurred in approximately 5% of everolimus-treated patients [16]. The reporting odds ratio for hyperglycemia with everolimus was 8.56, and a case of DKA has been reported [17, 18]. The present case had previously been treated with everolimus (administered once daily on consecutive days), during which hyperglycemia was mild.

Capivasertib caused grade 3/4 hyperglycemia in 2.3% of patients; however, glycemic fluctuations observed with isCGM appear more pronounced with capivasertib than they do with alpelisib [13, 14]. The hyperglycemic period corresponded to capivasertib’s Tmax (1 h) and half-life (10 h) [19]. Hyperglycemia occurring only during the treatment period suggests a pharmacodynamic correlation and reversible effect of capivasertib on glucose metabolism. In the CAPItello-291 trial, fasting glucose levels were assessed on days 1 and 15 of the first cycle and then every 4 weeks. Because routine laboratory monitoring typically occurs at the end of the capivasertib-free period, transient hyperglycemia during active treatment may be under-recognized. In addition, reliance on HbA1c measurements may underestimate the extent of hyperglycemia. Physicians should be aware of potential glycemic spikes during dosing days. Patients at risk for hyperglycemia or those with rising blood glucose levels or HbA1c should consider additional glucose monitoring during treatment. During capivasertib therapy, individualized interventions—such as targeted treatment only on dosing days—may be necessary.
